# Using the Decrease in Trauma Admissions During the COVID-19 Pandemic to Evaluate Compliance With Stay-at-Home and Social Distancing Guidelines

**DOI:** 10.7759/cureus.14444

**Published:** 2021-04-12

**Authors:** Paras Savla, James Wiginton, Taha M Taka, Tye Patchana, Ronit Farahmandian, Saman Farr, James A Berry, Mark Krel, Kevin Ray, Sarah Petrova, Jason Duong, Dan E Miulli

**Affiliations:** 1 Neurosurgery, Riverside University Health System Medical Center, Moreno Valley, USA; 2 Neurosurgery, University of California Riverside, Riverside, USA; 3 School of Medicine, California University of Science and Medicine, Colton, USA; 4 Neurosurgery, California University of Science and Medicine, Colton, USA; 5 Neurological Surgery, Arrowhead Regional Medical Center, Colton, USA; 6 Neurosurgery, Arrowhead Regional Medical Center, Colton, USA

**Keywords:** covid-19, motor vehicle collison, public health problems, trauma patients, trauma hospital, coronavirus pandemic, trauma, global pandemic

## Abstract

Introduction

The coronavirus disease 2019 (COVID-19) virus was declared a pandemic on March 10, 2020 by the World Health Organization (WHO) and has massively burdened healthcare systems with cases exponentially rising throughout the United States and the rest of the world. Since implementing precautions to reduce the spread of this disease, emergency departments have seen a decrease in the number of traumas. By evaluating the differences in the number of trauma admissions in different subgroups of patients, we can assess where to target messaging to increase compliance with these precautions. In this study, we aim to analyze the effect of the COVID-19 pandemic on trauma admissions.

Methodology

This was a retrospective review of the trauma database at our institution, a level 2 trauma center in Southern California, to assess the impact of COVID-19 on the number of traumas. The inclusion criteria were patients activated as traumas, regardless of age. Patients were excluded from the study if they did not have complete information in the trauma database. Data were stratified by date into two groups: (a) COVID period (January to April 2020) and (b) pre-COVID period (January to April 2019). The primary endpoint of this study was to determine whether there was a significant change in the number of patients presenting as trauma during the COVID-19 pandemic. This difference was analyzed and divided into subgroups based on age and trauma type.

Results

In our review, an average of 279 patients per month presented as trauma from January to April in 2019, and an average of 222 patients per month presented as trauma from January to April 2020 (p = 0.049). We found a significant decrease in the number of patients presenting with the chief complaint of fall and vehicular accident, but a nonsignificant difference in patients presenting as assaults or pedestrian accidents. There was also a significant decrease in the number of traumas in the 18-39 and 65+ age groups and a nonsignificant decrease in the 40-64 age group. It was also noted that the number of trauma admissions in May 2020 increased to 253 compared to 269 in 2019. This increase was most notable in the 18-39 and 40-64 age groups.

Discussion

As seen in the data, the most notable year-over-year difference was seen in March and April. In California specifically, a stay-at-home order was set in place in March, which was in conjunction with the WHO’s declaration of a pandemic. An interesting finding was the significant decrease in patients with traumas in the age groups of 18-39 and 65+ from 2019 to 2020. There was a smaller, nonsignificant decrease in patients aged 40-64. This would be a good group to target with future messaging to increase compliance with health advisories. There was also a notable increase in the number of traumas in May 2020, signaling an end to the cooperation of the majority of people, also indicating that further measures needed to be enacted in all groups.

Conclusions

COVID-19 has disrupted social structures worldwide. As the pandemic continued, even the observers of stay-at-home and social distancing measures, the 18-39 age group, became fatigued with the guidelines and ventured out into the warming weather and summer activities. This difference in trauma admission due to COVID-19 between subsequent years can highlight the behavioral changes in our patient population and can be further extrapolated to target additional messaging to help reduce the spread of COVID-19.

## Introduction

The coronavirus disease (COVID-19) virus was declared a pandemic on March 10, 2020 by the World Health Organization (WHO) and had since massively burdened healthcare systems with cases exponentially rising throughout the United States and the rest of the world. In efforts to slow the spread and avoid overwhelming hospitals, many states implemented and encouraged quarantining measures, closure of non-essential business, and a higher proportion of remote work. With the establishment of the “new normal,” new trends were seen with emergency department (ED) admission rates. A notable trend was the decrease in­­ automobile traffic volume that was seen nationally [1], which led to reports of fewer traffic accidents throughout states such as Florida and Missouri [2], and internationally in Turkey and Australia [1,3,4]. Concurrently, the lockdowns have led to decreased number of trauma cases [5-7] by 57.4% [8], contributing to a decrease in the volume of neurosurgical care by 40% [9-11].

In addition to a decrease in the number of vehicle accidents, it was shown­­ that the number of people seeking healthcare for minor injuries diminished after the COVID-19 pandemic [7]. A Centers for Disease Control and Prevention study comparing the ED ad­­­­mission rates with the admission rates of 2019 determined a decline across adult and pediatric populations. The decline in admission rates was most significant in minor injuries like musculoskeletal pain, sprains and strains, and superficial injuries, among other complaints/injuries [12]. The reason for this decline is likely due to the public awareness of the massive COVID patient influx in hospitals and the effort of patients with minor injuries to not overwhelm the hospital system and conserve finite hospital resources, as well as a fear of contracting COVID-19 during the hospital visit. In a study comparing the degree of decline between motor vehicle collision (MVC) trauma visits and non-MVC trauma visits, a larger decrease in MVC visits (80.5% decline) was seen in comparison to non-MVC trauma visits (45.1%) [8].

It is important to note that while the lack of MVC trauma visits is clearly explained by the decrease in traffic volumes, the decrease in non-MVC visits is more concerning. As previous studies have discussed, the decrease in hospital admission rates may signal an exacerbation of future chronic patient conditions and mortality rates [13,14]. A study of five states first to experience the COVID-19 surge witnessed increased mortality rates for conditions such as diabetes, heart disease, and Alzheimer’s disease [14].

However, given the severe social and economic impact of stay-at-home orders along with social distancing guidelines, not all subgroups within the population follow these orders to the same extent. As noted in prior studies, certain age groups are more likely to have violated these orders [15]. People are either unable to follow these strict rules or decide they are not necessary; the reasoning varies from person to person. It then becomes important to identify these specific groups so messaging can be tailored to these subsets of the population. By having these groups increase their compliance, the pandemic can be brought under better control until widespread vaccination allows for the development of herd immunity.

In this study, we will attempt to analyze the effect of the COVID-19 pandemic on trauma admissions at our level 2 trauma center to gain a better grasp of the economic and healthcare impact that traffic accidents and other injuries can cause. We also look more closely at age-related trends (especially of the elderly population) and specific mechanisms of injury to better outline groups needing additional targeting to increase compliance with stay-at-home and social distancing guidelines.

## Materials and methods

This was a retrospective review of the trauma database at our institution, a level 2 trauma center in Southern California, to assess what impact the COVID-19 virus had on the number of traumas. The inclusion criteria were patients activated as traumas, regardless of age. Patients were excluded from the study if they did not have complete information in the trauma database.

Demographic data including age, gender, and trauma type (falls, vehicular injury, assault, pedestrian, and all others) were recorded and collected, along with the date of presentation to the hospital. Data were stratified by date into two groups: (a) COVID period (January to April 2020) and (b) pre-COVID period (January to April 2019). We also included interesting data from May, which is the first month after the six-week stay-at-home order would have initially been lifted.

The primary endpoint of this study is to determine whether there was a significant change in the number of patients presenting as trauma during the COVID-19 pandemic. This difference was analyzed and divided into subgroups based on age and trauma type.

Statistical analysis was performed using the Student’s t-test to compare the sample means and assess for any differences in patient volume across the two time periods.

## Results

In our review, an average of 279 patients per month presented as trauma from January to April in 2019 and an average of 222 patients per month presented as trauma from January to April 2020. As seen in Table 1, this difference was statistically significant with a p-value of 0.049. Looking at the individual months, there was a decrease in the total number of traumas year over year in all months except February. The starkest difference was in the months of March and April. By subdividing the traumas into four categories (falls, vehicular accidents, assault, and pedestrian accidents), we see a significant decrease in the number of patients presenting with the chief complaint of fall and vehicular accident, but a nonsignificant difference in patients presenting as assaults or pedestrian accidents. These results are outlined in Table 1.

**Table 1:** Trauma admissions from January to April in 2019 and 2020 based on the mechanism of injury.

	Jan ‘19	Feb ‘19	Mar ‘19	Apr ‘19	Average	Jan ‘20	Feb ‘20	Mar ‘20	Apr ‘20	Average	P-Value
Falls	101	79	117	98	98.75	81	83	88	61	78.25	0.05
Vehicle	102	91	118	116	106.75	88	79	82	65	78.5	0.03
Assault	31	24	31	37	30.75	35	22	40	27	31	0.48
Pedestrian	22	14	16	12	16	19	18	10	9	14	0.21

Patients were stratified into three age groups for further analysis: (a) 18-39, (b) 40-64, and (c) 65+. There was a significant decrease in the number of traumas in the 18-39 and 65+ age groups and a nonsignificant decrease in the 40-64 age group (Table 2).

**Table 2:** Trauma admissions from January to April in 2019 and 2020 based on age groups.

Age	Jan ‘19	Feb ‘19	Mar ‘19	Apr ‘19	Average	Jan ‘20	Feb ‘20	Mar ‘20	Apr ‘20	Average	P-Value
18-39	119	92	140	124	118.75	108	86	110	77	95.25	0.04
40-64	68	62	83	75	72	68	72	65	49	63.5	0.19
65+	80	57	74	80	72.75	56	56	55	45	53	0.03

It is also noted that the number of trauma admissions in May of 2020 increased to 253 compared to 269 in 2019. This increase is most notable in the 18-39 and 40-64 age groups, increasing to 136 and 74, respectively.

## Discussion

COVID-19 is the first virus in modern history to cause such a major disruption to the way people socialize and interact with one another. Due to local, statewide, and national social distancing guidelines and lockdowns, fewer people are participating in activities that led to injuries requiring hospitalization [2,16]. Several studies from around the world have described the decreased trauma rates due to these restrictions [3,5,7,8].

The specific decreases noticed at our institution can also be linked directly to the impact of COVID-19. As seen in the data, the most notable year over year difference was seen in March and April. In California specifically, a stay-at-home order was set in place in March, which was in conjunction with the WHO’s declaration of a pandemic. There was still a notable decrease in January, possibly due to many more people having COVID-19 than was detected and reported, which inherently limited their activity preventing them from falling and driving. However, as word of a global disease began racing through the airwaves in the United States in late January, the resultant behavior may have been to initially limit activity. It is interesting to note that in February, there was no change in the number of traumas from 2019 to 2020. This could be due to low reported numbers of cases and deaths and the perceived lack of danger in the early stages of the pandemic, which has been found to be the strongest predictor of social distancing behavior [17].

When categorizing traumas into specific types including falls, vehicular accidents, assaults, and pedestrian accidents, a significant decrease in the number of falls and vehicular accidents was identified. The decrease in falls may have a twofold explanation. For one, patients have been avoiding the hospital due to concerns of being infected with COVID-19, as seen in an overall decrease of patients with a variety of pathologies [13]. Also, patients were limited in their access to activities outside the home, reducing the number of activities that could result in falls. As falls in elderly patients usually occur at home, a decrease may be due to additional family support available at home due to the stay-at-home orders. The decrease in vehicular accidents is consistent with the decrease in traffic and therefore traffic accidents seen around the world [1,2]. As such, fewer patients would be brought to the hospital as trauma activations.

An interesting finding was the significant decrease in patients with traumas in the age groups of 18-39 and 65+ from 2019 to 2020. There was also a decrease in the average number of monthly patients in the 40-64-year-old age group, but it was not statistically significant. This unique finding in the 40-64 age group is consistent with Canning et al. who found an increased odds ratio in patients who stated in a survey that they did not stay home. People in the age groups of 40-49, 50-59, and 60-69 stated they left their homes in the previous 24 hours while people in the age groups of 30-39 and >70 had decreased odds ratio of leaving their homes [15]. Simply stated, the age groups, 40-69, were more likely to have violated the order. It is possible that people from the ages 18-39 took advice and guidance from medical professionals with urgency to stay at home and social distance and those 65+ followed the guidelines due to increased risk from serious illness from COVID-19. This finding is especially important in directing further efforts in these age groups to promote safer social interactions to help reduce the spread of the virus.

Another notable finding is the increase in trauma admissions seen in May 2020. These began to reach 2019 levels, although they were still slightly lower. This could be related to a number of reasons. Having already been under quarantine orders for six weeks and facing significant social and economic consequences, people may have become weary of continuing social distancing. In addition, several social holidays, including Cinco de Mayo, Memorial Day, and the start of the summer season, may have led to more social gatherings outside of the home. It is also important to note that the largest increase in traumas in May occurred in the 18-39 age group. The members of this group, having followed guidelines for the previous two months, may represent the group that became disenchanted with these limitations the fastest. Based upon March and April decreased trauma admissions, the 18-39 age group more likely followed stay-at-home and social distancing guidelines; therefore, it may be important to reinforce the importance of these guidelines to encourage continued compliance targeted at the specific age group and response to the current circumstances. The 40-64 age group also had an increase in trauma admissions in May 2020, again demonstrating that this group is least likely to have been following the guidelines. This again exemplifies that this age group needed to be further educated to increase compliance.

One limitation of our study is that it is a single institutional experience, which may not be representative of a statewide, national, or global population. Although the data are consistent with previously published studies, it would be important to conduct further studies across multiple institutions within a community in various regions to further characterize the impact of COVID-19. Another limitation is the broad categories of trauma used in the trauma database. It is impossible to further delineate the specific causes of trauma, which could help identify more direct links to COVID-19 and the new guidelines created to combat the spread of the virus.

## Conclusions

COVID-19 has disrupted social structures worldwide. These disruptions can be directly seen in relation with trauma admissions at our institution. There was a significant decrease in trauma admissions in the months of January to April from 2019 to 2020. There was a decrease in falls and vehicular accidents in January 2020 compared to January 2019 possibly due to COVID-19 affecting the activities of the patient population prior to widespread detection and reporting. Initial reporting of low numbers of COVID-19 cases and deaths in February 2020 and the recovery from possible COVID-19 infections in January led to the return of personal activity and its resultant trauma. As COVID-19 cases began to rise in March and April, age groups limited their activity except the 40-64 age group. As the pandemic continued, even the observers of stay-at-home and social distancing, the 18-39 age group, saw increasing trauma admissions. This difference in trauma admission due to COVID-19 between subsequent years can highlight the behavioral changes in our patient population and can be further extrapolated to target additional messaging to help reduce the spread of COVID-19.
